# Identification of a *cis*-Acting Element Derived from Tomato Leaf Curl Yunnan Virus that Mediates the Replication of a Deficient Yeast Plasmid in *Saccharomyces cerevisiae*

**DOI:** 10.3390/v10100536

**Published:** 2018-09-30

**Authors:** Fangfang Li, Xiongbiao Xu, Xiuling Yang, Zhenghe Li, Xueping Zhou

**Affiliations:** 1 State Key Laboratory for Biology of Plant Disease and Insect Pest, Institute of Plant Protection, China Academy of Agricultural Sciences, Beijing 100193, China; elva1988@163.com (F.L.); xiulingyang@zju.edu.cn (X.Y.); 2 State Key Laboratory of Rice Biology, Institute of Biotechnology, Zhejiang University, Hangzhou 310058, China; xuxiongbiao1988@foxmail.com (X.X.); lizh@zju.edu.cn (Z.L.)

**Keywords:** tomato leaf curl Yunnan virus (TLCYnV), replication initiator protein (Rep), common region (CR), rolling circle replication (RCR), autonomous replication sequence (ARS)

## Abstract

Geminiviruses are a group of small single-stranded DNA viruses that replicate in the host cell nucleus. It has been reported that the viral replication initiator protein (Rep) and the conserved common region (CR) are required for rolling circle replication (RCR)-dependent geminivirus replication, but the detailed mechanisms of geminivirus replication are still obscure owing to a lack of a eukaryotic model system. In this study, we constructed a bacterial–yeast shuttle plasmid with the autonomous replication sequence (ARS) deleted, which failed to replicate in *Saccharomyces cerevisiae* cells and could not survive in selective media either. Tandemly repeated copies of 10 geminivirus genomic DNAs were inserted into this deficient plasmid to test whether they were able to replace the ARS to execute genomic DNA replication in yeast cells. We found that yeast cells consisting of the recombinant plasmid with 1.9 tandemly repeated copies of tomato leaf curl Yunnan virus isolate Y194 (TLCYnV-Y194, hereafter referred to as Y194) can replicate well and survive in selective plates. Furthermore, we showed that the recombinant plasmid harboring the Y194 genome with the mutation of the viral Rep or CR was still able to replicate in yeast cells, indicating the existence of a non-canonic RCR model. By a series of mutations, we mapped a short fragment of 174 nucleotides (nts) between the V1 and C3 open reading frames (ORFs), including an ARS-like element that can substitute the function of the ARS responsible for stable replication of extrachromosomal DNAs in yeast. The results of this study established a geminivirus replication system in yeast cells and revealed that Y194 consisting of an ARS-like element was able to support the replication a bacterial–yeast shuttle plasmid in yeast cells.

## 1. Introduction

Geminiviruses are plants single-stranded DNA viruses that are characterized by unique twinned icosahedral particles. The genome is composed of covalently closed circular single-stranded DNA of about 2.5–3.0 kb for monopartite geminiviruses, and about 4.8–5.6 kb for bipartite geminiviruses. According to the organization of their genomes, biological properties, type of insect vectors, and host ranges, the *Geminiviridae* are divided into nine genera (*Becurtovirus*, *Begomovirus*, *Curtovirus*, *Eragrovirus*, *Mastrevirus*, *Topocuvirus*, *Turncurtovirus*, *Capulavirus,* and *Grablovirus*) [1]. *Begomovirus* represents the largest genus, in which viruses are transmitted by the whitefly (*Bemisia tabaci*). Begomoviruses have two kinds of genome construction: bipartite, with a DNA-A component encoding all the proteins functions in virus replication, and the DNA-B component encoding the nuclear shuttle protein (NSP) and movement protein (MP) responsible for viral intracellular or cell to cell movement, respectively [2,3]. The monopartite begomoviruses contain the genome, just like the DNA-A component of bipartite begomoviruses, and are often associated with betasatellites, which are very important as they induce severe symptoms in crops [4]. The unique *βC1* open reading frame (ORF), encoded by the betasatellite complementary strand, is a pathogenicity determinant, and was reported to be a suppressor of both transcriptional gene silencing and post-transcriptional gene silencing [4,5,6].

Geminiviruses replicate in the nucleus of infected host cells by rolling-circle replication (RCR) [7,8]. The replication initiator protein (Rep), known as AC1/C1, is the major viral protein responsible for viral DNA replication. It is a multifunctional protein with site-specific nicking and ligase activity, helicase, site-specific DNA binding, and ATPase activity [9,10,11,12,13,14]. Rep regulates its own transcription by binding to iterative sequences (iterons) located within the common region (CR) between the TATA box and the AC1/C1 transcription start site to promote viral DNA replication [15,16,17,18,19]. Besides Rep, the AC3/C3 protein, known as the replication enhancer protein (REn), can provide a suitable cellular environment for viral DNA replication [20,21], and AC5, a pathogenicity determinant, also contributes to the viral genome replication [22,23]. 

Rep itself does not possess DNA polymerase activity but it relies on host DNA replication machinery for viral multiplication [24]. Rep can interact with host factors and recruit them to replicate the viral genome, such as retinoblastoma-related proteins (RBRs), which negatively regulate the cell cycle [25,26,27]. In a normal cell cycle, RBR interacted with the E2F family of DNA-binding transcription factors to form a complex, blocking E2F to transcribe the genes related to DNA replication [28]. Meanwhile, Rep interacted with RBR to release E2F from the RBR-E2F complex during geminivirus infection, leading to the activation of genes related to DNA replication [27,29,30]. The plant proliferative cell nuclear antigen (PCNA) interacted with Indian mungbean yellow mosaic virus (MYMIV) Rep, and repressed Rep’s site-specific nicking-closing activity and ATPase function [31]. However, begomovirus Rep hijacked replication factor C (RFC), preventing PCNA binding to viral dsDNA intermediates to facilitate viral DNA replication [32]. In addition to these Rep-interacting proteins, many other host factors were also found to be involved in DNA proliferation, including Replication protein A (RPA) [33], RAD54 [34], RAD51 [35], MCM2 [36], Methyltransferase (MET1), and Chromomethylase 3 (CMT3) [37].

Yeast is a model eukaryotic cell that has been used extensively to study the roles of individual genes in cellular processes involved in positive RNA virus replication and transcription owing to its small genome size and available single-gene-knockout (YKO) library [38,39]. Many host factors associated with tomato bushy stunt virus replication have been identified based on a library of temperature-sensitive mutants [40]. It has been reported that MYMIV was able to replicate in *Saccharomyces cerevisiae* [22], which represented the first yeast system supporting geminivirus replication. Based on the similar principle, we tested nine other geminiviruses in addition to MYMIV to find out whether they can replicate in yeast. However, we found that only tomato leaf curl Yunnan virus isolate Y194 (TLCYnV-Y194, hereafter referred to as Y194) was capable of replicating in yeast cells. Interestingly, the mutation of viral replication-related proteins or elements in the recombinant plasmid (a bacterial–yeast shuttle plasmid deleted of an autonomous replication sequence (ARS)) with the Y194 genome was still able to replicate in yeast cells, suggesting the existence of a non-canonic RCR model. We further identified a functional fragment of 174 nucleotides (nts) located in between V1 and C3 containing an ARS-like element in yeast cells, which functionally substituted the yeast ARS element. This is the first report of a geminivirus genome sequence including an ARS-like element to support the stable replication of viral DNA and the recombinant plasmid in yeast cells. We therefore concluded that this cis-acting element present in the Y194 genome is possibly able to be recognized by cellular factors that in turn support the replication of the viral DNA and the recombinant plasmid as an extrachromosomal element in yeast.

## 2. Materials and Methods

### 2.1. Yeast Stains 

The yeast strains BY4741, SC1, and W303-1B (MATa his3Δ1 leu2Δ0 met15Δ0 ura3Δ0) were stored in our laboratory.

### 2.2. Plasmid Construction

The original pRS316 plasmid (a yeast centromere vector with a URA3 marker and a multiple cloning site from pBluescript) was obtained from SnapGene, which allows the transformant to replicate and grow on Synthetic Complete (SC) media lacking uracil (SC/–uracil). To remove the ARS of the plasmid pRS316, the primer pair dARSH4/NheI/F and dARSH4/NheI/R was used. The PCR products were digested with NheI and then ligated using T4 DNA ligases (NEB^®^, Beijing, China). The resulting plasmid was obtained and confirmed by sequencing. It is hereafter referred to as dpRS316, and fails to replicate in yeast cells owing to the deficiency of the ARS. 

The plasmid dpRS316-Y194-1.9A was constructed as follows: a 0.9A copy of Y194 genomic DNA was amplified by using the infectious clone plasmid of Y194 [41] as a template with primer pair Y194/XhoI/F and Y194/BamHI/R. The PCR products were purified using the AxyPrep DNA Gel Extraction Kit (Axygen Scientific Inc.^®^, Union City, CA, USA) following the manufacturer’s instructions and then digested by XhoI and BamHI. The product was ligated to dpRS316 treated with the same enzymes to produce dpRS316-Y194-0.9A. A full-length copy of Y194 genomic DNA was amplified by using primer pair Y194/BamHI/F and Y194/BamHI/R, then digested with BamHI and ligated to BamHI-digested dpRS316-Y194-0.9A to obtain the plasmid dpRS316-Y194-1.9A, which was further confirmed by sequencing. Another nine recombinant plasmids containing at least 1.2 copies of geminivirus DNAs, including tomato yellow leaf curl China virus isolate Y10 (TYLCCNV-Y10), MYMIV, tomato yellow leaf curl virus isolate SH2 (TYLCV-SH2), tobacco leaf curl Yunnan virus isolate Y143 (ToLCYnV-Y143), tomato yellow leaf curl Thailand virus isolate 72 (TYLCTHV-Y72), clerodendrum golden mosaic China virus (CLGMCNV), clerodendrum golden mosaic Jiangsu virus (CLGMJSV), cotton leaf curl Multan virus isolate 37 (CLCuMV-37), and African cassava mosaic virus (ACMV) were obtained in similar strategies, generating dpRS316-TYLCCNV-Y10-1.7A, dpRS316-MYMIV-1.65A, dpRS316-TYLCV-SH2-1.22A, dpRS316-ToLCYnV-Y143-1.9A, dpRS316-TYLCTHV-Y72A-1.9A, dpRS316-ClGMCNV-1.4A, dpRS316-CLGMJSV-1.43A, dpRS316-CLCuMV-G37A-1.9A, and dpRS316-ACMV-1.24A, respectively.

The point mutants of dpRS316-Y194-0.9A in different positions of the Y194 genome were constructed as follows: the 0.9A fragment of Y194 genome was obtained by digestion with XhoI and BamHI restriction endonuclease and was ligated to the pMD-18T vector (TAKARA^®^, Tokyo, Japan) to obtain pMD18T-Y194-0.9A. A series of mutations were introduced in the CR, C1 start codon, the Y103F of C1, C3 start codon, and the C4 start codon using the primer pairs in Table S1. All mutants were confirmed by restriction enzyme digestion and sequencing, and then subcloned into dpRS316 vector to produce the mutant plasmids, hereafter referred to as dpRS316-Y194-0.9Am1, dpRS316-Y194-0.9Am2, dpRS316-Y194-0.9Am3, dpRS316-Y194-0.9Am4, and dpRS316-Y194-0.9Am5, respectively. 

In order to determine the much shorter fragment of Y194 that acts as an ARS-like element, a series of mutants (d-Y194-M1 to d-Y194-M20, with the exception of d-Y194-M7 and d-Y194-M8) containing different parts of the Y194 genome were constructed with the primer pairs in Table S1. The constructs of d-Y194-M7 and d-Y194-M8 were obtained by cutting d-Y194-1.9A using BamHI and EcoRI, which generated two fragments including Y194 genome sequences (one fragment is 1664 bp, and the other one is 1117 bp) and were ligated to the dpRS316 vector, respectively.

The resulting plasmid d-CEN with the lack of a centromere (CEN)-like element in the backbone of the plasmid pRS316 was constructed using this primer pair: dCEN/NheI/F and dCEN/NheI/R. Recombinant plasmids d-CEN-Y194-0.9A and d-CEN-Y194-1.9A were constructed in a similar strategy with dpRS316-Y194-0.9A and dpRS316-Y194-1.9A, respectively.

### 2.3. Yeast Transformation

The transformation of yeast cells with the plasmids was performed as previously described [22]. Single-stranded DNA was used as the carrier DNA to enhance the efficiency of the plasmid-mediated transformation. Yeast cells from three yeast strains—W303-1B, SC1, and BY4741 (MATa his3Δ1 leu2Δ0 met15Δ0 ura3Δ0)—were transformed with the above plasmids which contain the Ura3 marker, and the colonies were scored on SC/–uracil plates. 

### 2.4. Plasmid Stability Assay

The plasmid stability experiment was carried out as described previously [42,43]. The W303-1B yeast cells transformed with d-CEN-Y194-0.9A, d-CEN-Y194-1.9A, d-CEN (negative control), and pRS316 (positive control) were first cultured on SC/–uracil media for 5 days (d), then transferred into SC liquid media to allow growth for 90 h. Pipetted 10 μL of cultures and diluted by 10-fold, then spotted on SC or SC/–uracil solid media. In order to determine the percentage of cells possessing plasmid, the numbers of cultures were counted on SC and SC/–uracil plate after 5 d of growth. The percentage of cells retaining plasmid was calculated by the ratio of the number of colonies present on the SC/–uracil versus the SC plate.

### 2.5. Southern Blot Assay

The W303-1B yeast cells transformed with dpRS316-Y194-0.9A, dpRS316-Y194-1.0A, dpRS316-Y194-1.9A, and pRS316 (positive control) were firstly cultured on SC/–uracil plates for 3 d, then transferred into SC/–uracil liquid media to allow growth for 60 h. Then the replicated yeast plasmids from yeast cells transformed with the above plasmids were extracted for Southern blot assay. To test whether there existed viral genome DNAs in dpRS316-Y194-0.9A, dpRS316-Y194-1.0A, and dpRS316-Y194-1.9A-transformed yeast cells, the shorter DNA fragments from yeast cells transformed with the above plasmids were separated, enriched, or treated with exonuclease I for Southern blot analysis.

## 3. Results

### 3.1. The Recombinant Plasmid Consisting of 1.9 Tandem Copies of the Y194 Genome Can Replicate in Yeast

**Figure 1:**
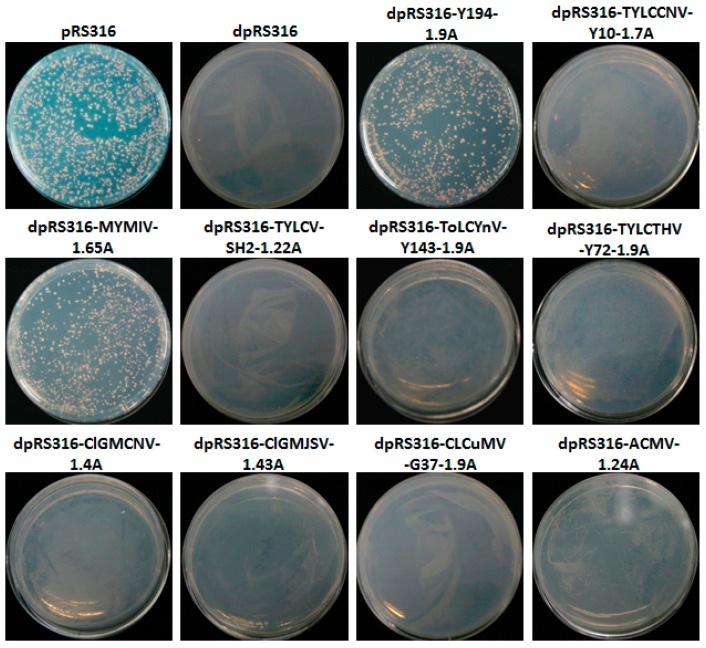
Screening of geminivirus replication using a deficient yeast plasmid in yeast cells. *Saccharomyces cerevisiae* W303-1B cells were transformed with pRS316 (the positive control plasmid), dpRS316 (the negative control plasmid), dpRS316-Y194-1.9A, dpRS316-TYLCCNV-Y10-1.7A, dpRS316-MYMIV-1.65A, dpRS316-TYLCV-SH2-1.22A, dpRS316-ToLCYnV-Y143-1.9A, dpRS316-TYLCTHV-Y72A-1.9A, dpRS316-CLGMCNV-1.4A, dpRS316-CLGMJSV-1.43A, dpRS316-CLCuMV-G37-1.9A, or dpRS316-ACMV-1.24A. Photographs were taken at 4 days (d) post transformation.

**Figure 2:**
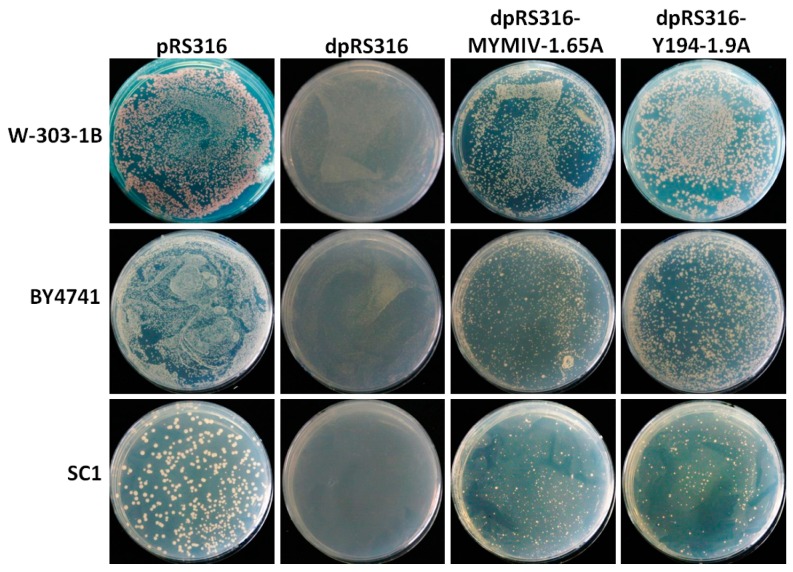
The recombinant plasmid with MYMIV or Y194 DNA could replicate in three types of yeast strains (W303-1B, BY4741 and SC1). The indicated yeast cells (W303-1B, BY4741 or SC1) were transformed with pRS316, dpRS316, dpRS316-MYMIV-1.65A, or dpRS316-Y194-1.9A, individually. Photographs were taken at 5 d after transformation.

Geminivirus infectious clone containing a tandem of more than 1.2 copies of the genomic DNA with two CRs is required for efficient infection in plants. To construct a yeast shuttle plasmid that contains the geminivirus genomic DNA to replicate in *S. cerevisiae*, a deficient yeast shuttle vector pRS316 (dpRS316) which lacks a 359-base pair (bp) fragment containing the ARS activity was obtained firstly. The detailed information of dpRS316 was described in Section 2.2 and Supplementary Materials Figure S1. This dpRS316 plasmid was able to survive in *Escherichia coli* in the Lysogeny broth (LB) media containing ampicillin but not in yeast cells in SC/–uracil yeast nutrition selection media owing to the lack of replication activity in yeast cells. The dpRS316 served as the base vector for the construction of recombinant plasmids with 10 geminiviruses’ genomic DNA, including Y194, TYLCCNV-Y10, MYMIV, TYLCV-SH2, ToLCYnV-Y143, TYLCTHV-Y72, CLGMCNV, CLGMJSV, CLCuMV-G37, and ACMV. The schematic diagrams of these constructs were shown in Figure S1. The resultant plasmids were transformed into yeast strain W303-1B cells, and yeast cells transformed with the original plasmid pRS316 and the ARS-deficient plasmid dpRS316 were used as positive controls and negative controls, respectively. All transformants were plated on a SC/–uracil yeast nutrition selection plate (Figure 1). Because of the lack of the ARS, the yeast cells transformed with the ARS-deficient plasmid dpRS316 failed to grow on the SC/–uracil yeast nutrition selection plate, but the yeast cells transformed with the original pRS316 were able to grow on this selective plate (Figure 1). Among these recombinant plasmids including geminivirus sequences, only the recombinant plasmids dpRS316-Y194-1.9A and dpRS316-MYMIV-1.65A can replicate in W303-1B yeast cells (Figure 1). Further, the other two yeast strains (SC1 and BY4741) were also used to confirm the replication of MYMIV and Y194 in yeast cells (Figure 2). Our data supported the previous result that the recombinant plasmid with bipartite begomovirus MYMIV DNA-A replicated in yeast cells [22], and found that the recombinant plasmid with monopartite begomovirus Y194 was also able to replicate in yeast cells.

### 3.2. A Non-RCR Model Supporting the Replication of the Recombinant Plasmid with Y194 DNA in Yeast Cells

**Figure 3:**
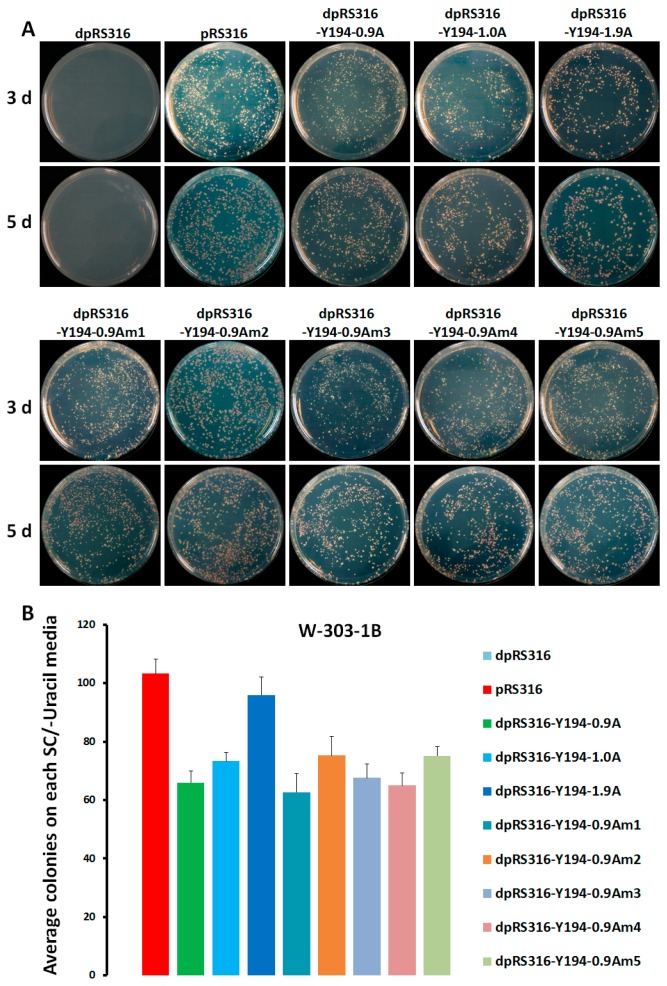
Replication of Y194-derived sequence carrying mutations in rolling circle replication (RCR)-related proteins and the *cis*-element in yeast cells. (**A**) W303-1B yeast cells were transformed with dpRS316, pRS316, dpRS316-Y194-0.9A, dpRS316-Y194-1.0A, dpRS316-Y194-1.9A, and the mutants of dpRS316-Y194-0.9A. Photographs were taken at 3 d and 5 d after transformation. (**B**) The average numbers of yeast colonies carrying the above plasmids were calculated. Three independent transformations with the indicated plasmids were performed, and the values represent the average numbers of yeast colonies (diameter > 2 mm) emerging on the Synthetic Complete (SC) media lacking uracil (SC/–uracil) after 5 d of transformation.

**Figure 4:**
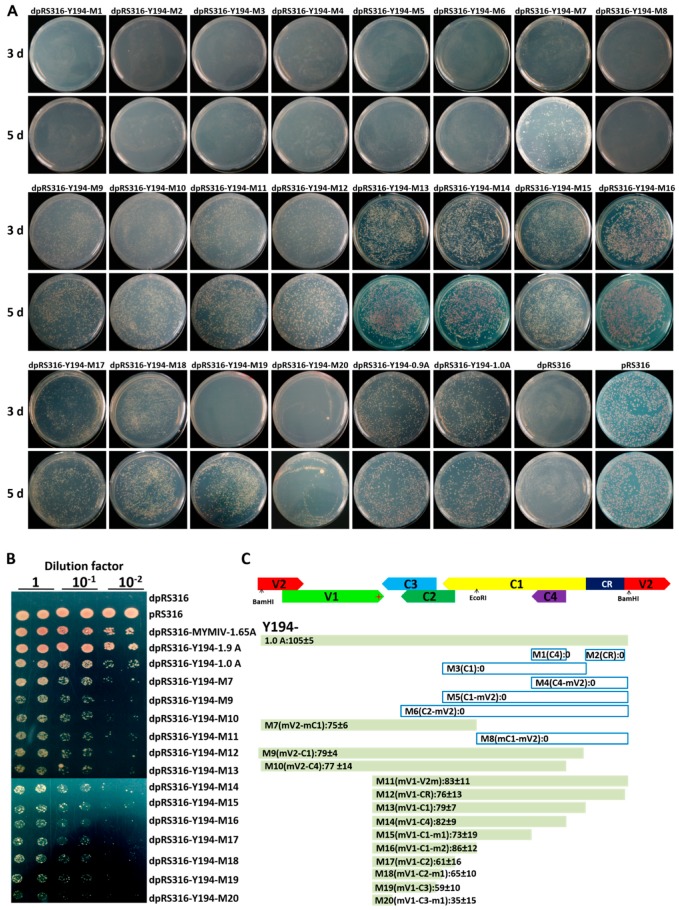
Identification of the yeast replication origin-like element sequences in the Y194 genome. (**A**) Recombinant plasmids dpRS316-(M1-M20) containing different fragments of Y194 viral genome DNA were transformed into W303-1B yeast cells, individually. Photographs were taken at 3 d and 5 d after transformation on the SC/–uracil plate. (**B**) The grown colonies of yeast cells transformed with pRS316, or dpRS316-Y194-1.0A,-M7, -M9-M16 from (**A**) were diluted on SC/–uracil. The yeast cells transformed with dpRS316-MYMIV-1.65A or dpRS316-Y194-1.9A were also used for positive controls, and yeast cells transformed with dpRS316 (**A**) as a negative control. (**C**) The diagrammatic sketch of different Y194 constructs and the average numbers of yeast colonies carrying the indicated plasmids. The genomic organization of Y194, including the common region (CR), the V2 open reading frame (ORF), the V1 ORF, the C3 ORF, the C2 ORF, the C1 ORF, and the C4 ORF located in the viral and complementary strands are indicated. The red star represents the position of ARS-like elements in Y194 genomic DNA sequences. Three independent transformations with the indicated plasmids (**A**) were performed, and the values represent the average numbers of yeast colonies (diameter > 2 mm) emerging on the SC/–uracil media after 5 d of transformation.

MYMIV has been reported to replicate well in yeast cells in a RCR model, for which replication was dependent on viral CR, replication-related proteins, and host factors [22]. Therefore, Y194 was used for further study. First of all, to confirm whether Y194 replicated in yeast using classical RCR or in other forms, the recombinant plasmid containing 0.9 copy or a full copy of the Y194 viral genome referred to as dpRS316-Y194-0.9A or dpRS316-Y194-1.0A was obtained. These recombinant plasmids, including dpRS316-Y194-0.9A, dpRS316-Y194-1.0A, and dpRS316-Y194-1.9A were transformed into yeast strain W303-1B to compare their growth activity. As shown in Figure 3, all these three recombinant plasmids-transformed yeast cells were able to grow well on SC/–uracil plates. The existence of Y194 DNA sequences in dpRS316-Y194-0.9A, dpRS316-Y194-1.0A, or dpRS316-Y194-1.9A-transformed yeast cells was confirmed by Southern blot analysis (Figure S2A). The expression of the intact Rep and the existence of a conserved stem-loop structure CR are the prerequisites for geminivirus RCR [3,24]). However, the recombinant plasmid dpRS316-Y194-0.9A only consisting of 0.9 copy of the Y194 genome, can replicate well as the construct dpRS316-Y194-1.9A in yeast cells, which prompted us to guess that RCR model is not required for Y194 to support the replication of the recombinant plasmid with viral DNA in yeast cells. In order to test this hypothesis, we constructed five mutants in the backbone of dpRS316-Y194-0.9A, including the invariant 9-mer sequence mutant (mutant TAATATTAC to TAATATCGC) (hereafter referred to as d-Y194-Am1), the null mutation of C1 (d-Y194-Am2), the Y103F point mutation to destroy the specificity-nicking activity of C1 protein (d-Y194-Am3), the null mutation of C3 (d-Y194-Am4), and the mutation of C4 (d-Y194-Am5). After confirmation by sequencing, these five mutants (dpRS316, pRS316, dpRS316-Y194-0.9A, dpRS316-Y194-1.0A, and dpRS316-Y194-1.9A) were transformed into W303-1B yeast cells. All the above plasmids were transformed into yeast cells in three independent times and calculated the average replication efficiency of each recombinant plasmid at 3 and 5 d post transformation. To our surprise, all these transformants can replicate as well as the dpRS316-Y194-0.9A-transformed yeast cells (Figure 4). These results indicate that the recombinant plasmid harboring the Y194 genome sequence is able to replicate in yeast cells but independent of its CR or its Rep. Therefore, we propose that the RCR model is dispensable for the replication of the recombinant plasmid with Y194 in yeast cells, and there may exist an element in Y194 genome that plays a role similar to an ARS in plasmid pRS316 to support the replication of the recombinant plasmid with Y194 in yeast cells.

### 3.3. A 174-nt Sequence Located in between the V1 and C3 ORF Includes an ARS-Like Element in the Y194 Genome

Based on our hypothesis that there may be a sequence element in Y194 function as the ARS of pRS316, we were engaged to identify a functional element that contributed to the replication of the recombinant plasmid including Y194 DNA in yeast cells. Then, we constructed a series of mutant plasmids using the primer pairs in Table S1, named d-Y194-M1 to d-Y194-M20, and each contained different parts of the Y194 genome. All these 20 recombinants, along with dpRS316, pRS316, dpRS316-Y194-0.9A, dpRS316-Y194-1.0A, and dpRS316-Y194-1.9A, were transformed into W303-1B yeast cells. At 3 and 5 d after transformation, we identified that yeast cells with dpRS316-Y194-M7/M9-M20 could grow in SC/–uracil plates (Figure 4A). To further confirm this result, we chose these grown yeast colonies from SC/–uracil plates and diluted them into a new SC/–uracil plate to compare their growth activity. As shown in Figure 4, we finally identified M20, containing a short sequence of 174 nt located in between V1 and C3 ORF (the position in the Y194 genome is from the 1027th nt to the 1201th nt), which is the functional cis-element which was able to execute the function of the ARS in yeast shuttle plasmid. In consistency with this fact, the ARS-like element ‘TTTTATGATTC’ (the ARS consensus sequences are A/T TTTAT A/G TTT A/T) in Y194 genomic sequences from the 1037th nt to the 1047th nt was identified (Figure S3). These data suggested that the recombinant plasmids including Y194 genomic DNA or its derivates are able to support the replication of the dpRS316 plasmid in yeast cells, possibly because of this ARS-like element.

### 3.4. Y194 Genome Lacks a Centromere (CEN)-Like Element

**Figure 5:**
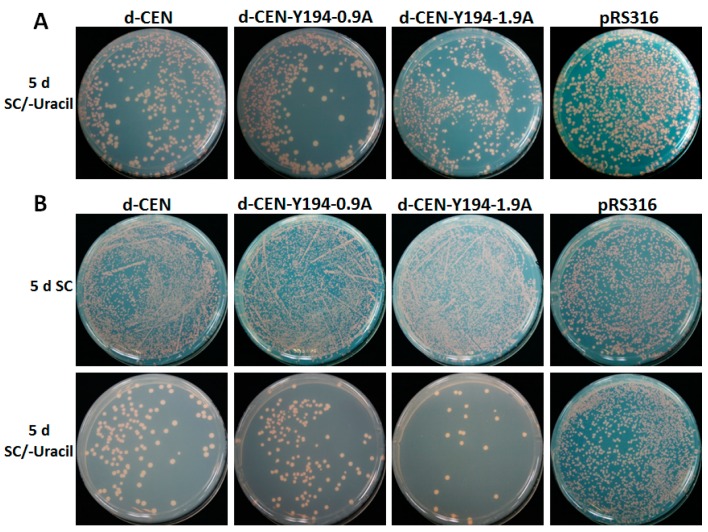

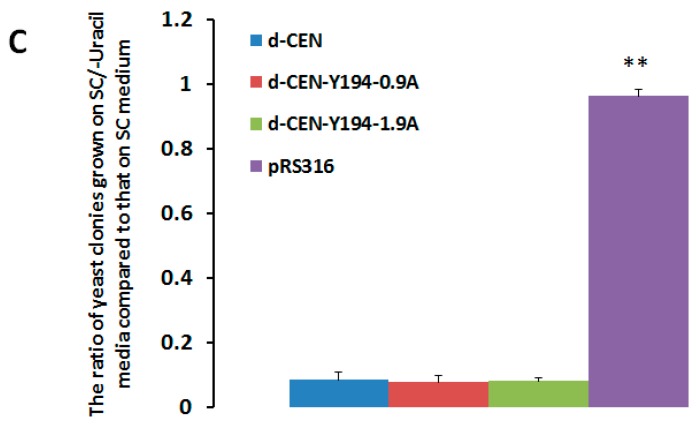
Identification of the centromere (CEN) element in Y194. (**A**) W303-1B yeast cells were transformed with d-CEN (negative control), d-CEN-Y194-0.9A, d-CEN-Y194-1.9A and pRS316 (positive control), individually. Photographs were taken at 5 d after transformation. (**B**) Yeast cells containing the indicated plasmids from (**A**) were cultured for 90-h generations in the SC liquid plate, and then were spread in the SC and SC/–uracil solid plate. Photographs were taken at 5 d after plating. (**C**) The ratio of yeast colonies grown on SC/–uracil media compared to that on the SC plate from (**B**). Three independent transformations with the indicated plasmids (**A**) were performed, and the values represent the average numbers of yeast colonies emerging on the SC/–uracil media compared to SC plate after 5 d of transformation. Values represent the mean ± standard deviation (SD). The data were analyzed using Student’s *t*-test and double asterisks denote a highly significant difference compared to d-CEN (** *p* < 0.01).

Human papilloma virus (HPV) genomic DNA not only contains an ARS-like element, but also possesses a CEN-like element, which can distribute the nascent DNA to the progeny cells equally during mitosis or meiosis to maintain the stabilization of viral genome [42,43]. Based on the fact that Y194 genomic DNA can autonomously replicate in W303-1B yeast cells, we wondered whether the Y194 genome also consisted of a CEN-like element to maintain the mitotic stability in yeast cells. For this purpose, we obtained another deficient plasmid of pRS316 (we called it d-CEN in this study), which lacks a CEN-like element but keeps the ARS sequence of pRS316. Then, the recombinant plasmids d-CEN-Y194-0.9A and d-CEN-Y194-1.9A were further generated based on the backbone plasmid, d-CEN. All these plasmids were transformed into W303-1B yeast cells respectively, and pRS316 plasmid was transformed as a positive control. A single colony of those transformed yeast cells was inoculated into SC liquid plate after 5 d of culture on SC/–uracil plates. After 90 h of incubation of yeast cells with the above plasmids in the SC liquid plate, 10 μL of the cultured cells were diluted into a final volume of 100 μL and then inoculated on the SC or SC/–uracil solid plate. The number of colonies in the SC or SC/–uracil solid plate was calculated after 5 d of culture. As shown in Figure 5, after 90 h of incubation in the SC liquid plate and a subsequent 5 d of culture on a SC/–uracil plate, there was no significant difference among d-CEN-Y194-0.9A, d-CEN-Y194-1.9A, and the negative control (d-CEN). The ratio (the average numbers of yeast colonies emerging on SC/–uracil media compared to that on SC plate after 5 d) of transformants of the positive control pRS316 was higher than that with the negative control dpRS316 (Figure 5). These data suggested that there was no CEN-like element in the Y194 genome.

## 4. Discussion

In this study, we used the deficient dpRS316 shuttle plasmid as a backbone to build a system which identified a cis-acting element for geminivirus replication in yeast cells (Figure 1, Figure 2, Figure 3 and Figure 4). Based on this system, we found that the recombinant plasmid with MYMIV (bipartite begomovirus) DNA or Y194 (monopartite begomovirus) DNA can replicate in yeast cells (Figure 1 and Figure 2). Raghavan et al. [22] demonstrated that two tandem copies of the MYMIV DNA-A can replicate in yeast cells, and we confirmed that 1.65 copies of MYMIV DNA-A were enough to support its viral DNA replication. The mutation of CR, Rep, AC5, other replication-related elements and viral proteins, and the deficiency of host factors inhibited MYMIV DNA-A replication [22,23], supporting that MYMIV replication occurred in the RCR model in yeast cells.

Y194 was firstly reported in 2013 by Xie et al. [41], and encodes a pathogenic factor C4, causing serious symptoms in tobacco and tomato plants [41]. We inserted 1.9-copies Y194 genomic DNA into the ARS absent dpRS316 vector and found that Y194 can replicate as well as the positive control pRS316 after transforming into W301-1B yeast cells (Figure 1 and Figure 2). We also constructed two recombinant plasmids, dpRS316-Y194-0.9A and dpRS316-Y194-1.0A, which consisted of a 0.9A or 1.0A copy of Y194 genomic DNA, respectively. To our surprise, all yeast cells transformed with recombinant plasmids containing 0.9A, 1.0A, or 1.9A-copies of Y194 genomic DNA were able to grow well on SC/–uracil-selective plates (Figure 3). Further, the conserved nonamer nucleotides TAATATTAC or Rep^Y103F^ were mutated, but yeast cells with these mutants still showed no obvious differences compared to with wild-type viral DNA (Figure 3). Thus, we concluded that Y194 may be able to multiply its genome in many forms in addition to a canonic RCR model in yeast cells. To determine whether the recombinant plasmids with Y194 genomic DNA could produce the viral genome in yeast cells, the separated and enriched short DNA fragments from the yeast cells transformed with dpRS316-Y194-0.9A, dpRS316-Y194-1.0A, dpRS316-Y194-1.9A, and pRS316 were analyzed using Y194 DNA probes by Southern blot. As shown in Figure S2B, dpRS316-Y194-1.9A, not dpRS316-Y194-0.9A, dpRS316-Y194-1.0A, or pRS316, could produce a specific DNA band of about 2.7 kb which was sensitive to exonuclease I (Figure S2C), suggesting that dpRS316-Y194-1.9A including two CRs probably produced the Y194 genome DNA in RCR. Combined with these results, it is possible that most of dpRS316-Y194-1.9A recombinant plasmids replicate in yeast cells by a viral DNA sequence, and a few of them produce the replication of the recombinant plasmids and viral genome in RCR model.

A sequence from HPV genome functioned as the ARS element and was able to autonomously replicate in yeast, which was important for viral DNA replication during viral life cycle [42,43]. We supposed that Y194 genomic DNA could possess an element acting as the ARS in yeast cells. Therefore, we selected different parts of Y194 genome and inserted these fragments into a dpRS316 vector to decide their replication ability in yeast cells (Figure 4)*.* We identified that a 174-nt fragment located in between V1 and C3 ORF possessed ARS activity and supported the recombinant plasmid to autonomously replicate in yeast cells (Figure 4). It is noteworthy that the ARS-like element ‘TTTTATGATTC’ was identified in this region (Figure S3), indicating that these sequences in Y194 genome serving as a functional element have ARS activity to mediate the replication of the dpRS316 plasmid in yeast cells. Compared to Y194, no ARS-like element was identified in these seven tested geminiviruses including MYMIV, ToLCYnV-Y143, TYLCTHV-Y72, CLGMCNV, CLGMJSV, CLCuMV-37 and ACMV, demonstrating that this ARS-like element in Y194 genome sequences is not universal in geminiviruses. However, this ARS-like element was also identified in TYLCCNV-Y10 and TYLCV-SH2, which share high sequence identity compared to the other seven geminiviruses with Y194. Although both of TYLCCNV-Y10 and TYLCV-SH2 have an ARS-like element, they could not support the replication of the dpRS316 plasmid in yeast cells (Figure 1)*.* Therefore, the ARS-like element in geminivirus sequences is required but not sufficient to mediate the replication of the ARS-deficient plasmid, suggesting that the sequences around ARS element are also important to influence the replication efficiency of this recombinant plasmid in yeast cells. The fact that more colonies emerged on dpRS-Y194-M16/M17/M18/M19-transformed yeast cells than that on dpRS-Y194-M16-transformed yeast cells supported this speculation (Figure 4). We also attempted to find whether there is a CEN-like element in Y194 genome (Figure 5). However, no CEN-like element was identified from Y194 genome in yeast cells (Figure 5).

To our knowledge, this is the first report about a short sequence from geminivirus DNA belonging to plant viruses that can serve as an ARS to support the stable replication of viral DNA and the ARS-deficient yeast plasmid in yeast. This short fragment containing an ARS-like element lies in between V1 and C3 of Y194 genome (precisely located in between the 1027th nt and 1201th nt) and is independent of CR or C1, which both have proved to be essential for geminivirus replication. However, there are still unclear and fascinating problems deserving further study to explore a cis-acting element for the geminivirus replication mechanism, particularly with respect to whether the ARS-like element from Y194 genome functions in plants as it does in yeast.

## 5. Conclusions

Ten recombinant yeast shuttle plasmids, including the Y194, TYLCCNV-Y10, MYMIV DNA-A, TYLCV-SH2, ToLCYnV-Y143, TYLCTHV-Y72, CLGMCNV, CLGMJSV, CLCuMV-G37, and ACMV genomes were constructed in this study. Only a recombinant plasmid consisting of MYMIV DNA-A or Y194 genomic DNA was found to be able to replicate in yeast cells. However, unlike MYMYV DNA-A, viral CR and replication-related proteins are not required for Y194 replication in yeast cells. A 174-nt sequence, including an ARS-like element located in between the V1 and C3 ORF of the Y194 genome, was further identified to support the stable replication of the recombinant plasmid with Y194 DNA in yeast cells. These findings establish a geminivirus replication system in yeast cells, clarifying the replication ability of different geminiviruses in yeast cells and the replication mechanisms of Y194. However, further studies are necessary to learn whether this ARS-like element located in Y194 genome DNA influences viral replication efficiency in plants, and determine the role of this ARS-like element in the viral life cycle. 

## Supplementary Materials

The following are available online at http://www.mdpi.com/1999-4915/10/10/536/s1, Figure S1: Architecture of original pRS316 and its derivates constructed in this study, Figure S2: Southern blot analyzed the existence of Y194 DNA sequences in dpRS316-Y194-0.9A, dpRS316-Y194-1.0A or dpRS316-Y194-1.9A-transformed yeast cells using Y194 DNA probe, Figure S3: Identification of a sequence in Y194 genome bearing similarity to ARS elements, Table S1: Sequences of primers used in this study.
