# Supplementary material for: Identification of a cis-Acting Element Derived from Tomato Leaf Curl Yunnan Virus that Mediates the Replication of a Deficient Yeast Plasmid in Saccharomyces cerevisiae
Source: Viruses. 2018 Sep 30;10(10):536. doi: 10.3390/v10100536 (PMC6213642; doi:10.3390/v10100536)
Supplement: Supplementary file 1 [file viruses-10-00536-s001.pdf]

viruses

MDPI

Supplementary Materials

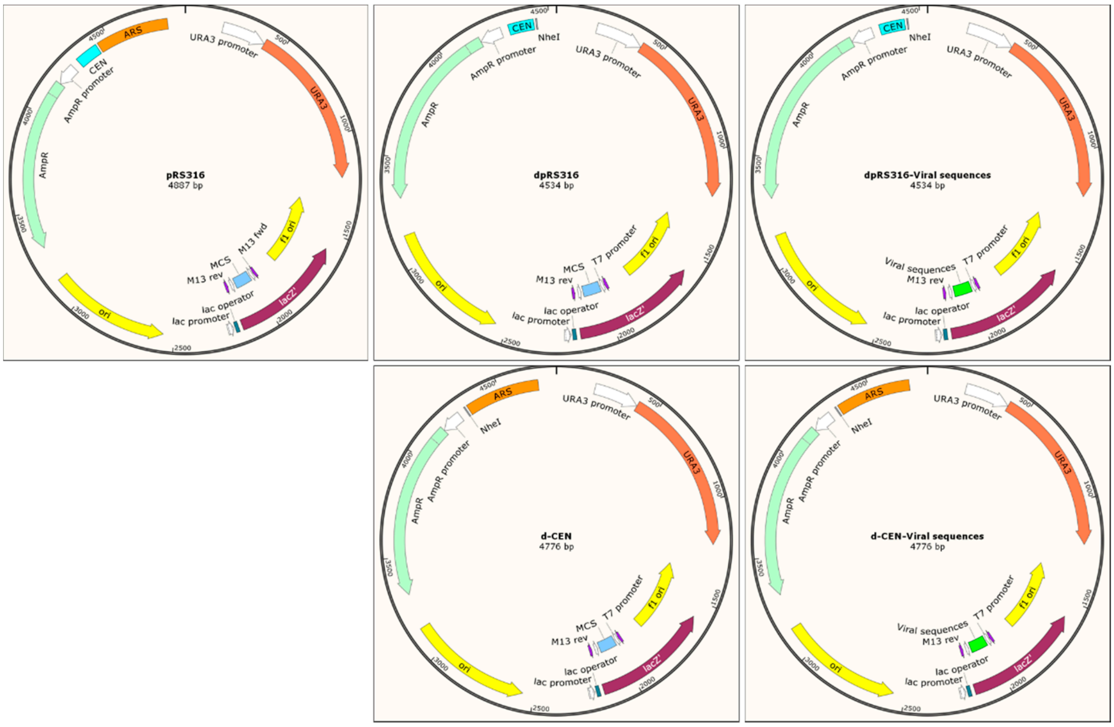

Figure S1. Architecture of original pRS316 and its derivates constructed in this study. The architecture of pRS316, dpRS316, pRS316-viral sequences, d-CEN, and d-CEN-viral sequences is depicted using 'SnapGene™'. The descriptions for these constructs in detail are shown in Section 2.2.

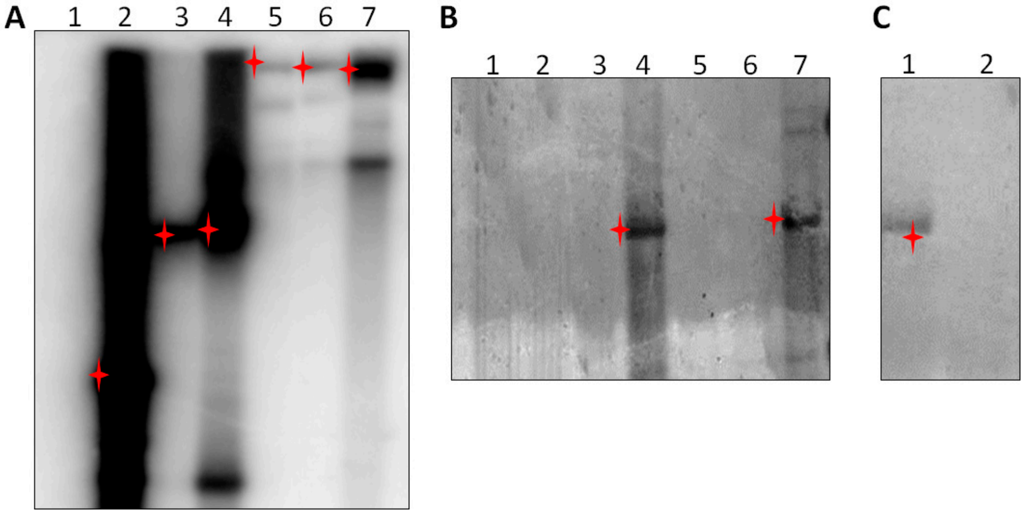

Figure S2. Southern blot analyzed the existence of Y194 DNA sequences in dpRS316-Y194-0.9A, dpRS316-Y194-1.0A, or dpRS316-Y194-1.9A-transformed yeast cells using a Y194 DNA probe. (**A**) Lane 1: negative control, extracted yeast plasmids from pRS316-transformed yeast cells; Lane 2/Lane 3/Lane 4: positive controls, 1.0-kb/2.4-kb/2.7-kb PCR products from Y194 DNA; Lane 5/Land 6/Lane 7: extracted yeast plasmids from dpRS316-Y194-0.9A/1.0A/1.9A-transformed yeast cells. (**B**) Lane 1: positive control, 2.7-kb PCR products from Y194 DNA; Lane 2/Lane 3/Lane 4: the short DNA fragments separated and enriched from dpRS316-Y194-0.9A/1.0A/1.9A-transformed yeast cells; Lane 5: negative control, the short DNA fragments separated and enriched from pRS316-transformed yeast cells. (**C**) The short DNA fragments separated and enriched from dpRS316-Y194-1.9A-transformed

yeast cells without (Lane1) or with exonuclease I (Lane2). The detailed information for Southern blot assay in this figure was described in Section 2.5. Red stars indicate the expected band sizes.

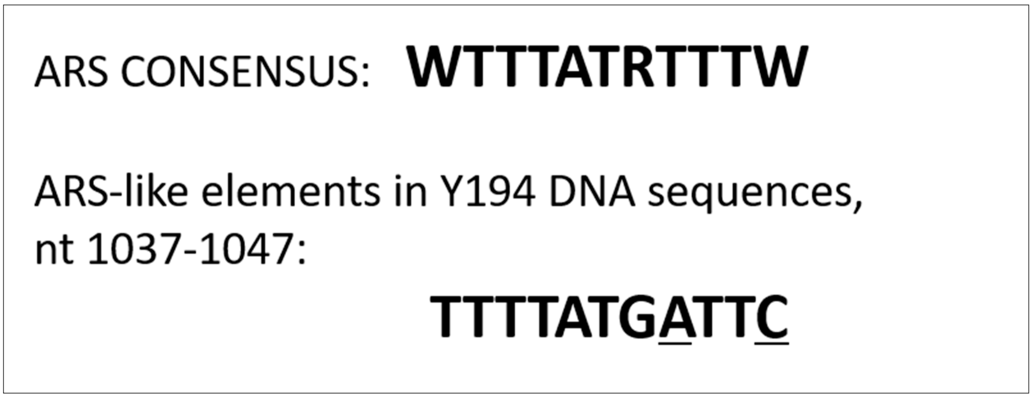

Figure S3. Identification of a sequence in Y194 genome bearing a similarity to ARS elements. ARS consensus sequences and ARS-like elements in Y194 genomic DNA sequences are shown. The nucleotide (nt) position information of this sequence is indicated to the left. Mismatches with the consensus sequences are underlined. W: A or T, R: A or G.

Table S1. Sequences of primers used in this study.

**Primers**	**Sequences (5′-3′)**	**Comments**
**dARSH4/NheI/F**	CGCGGCTAGCAGTTACAGGCAAGCGATCCGT	The NheI site is underlined
**dARSH4/NheI/R**	CGCGGCTAGCCTAGAGTCTTTTACATCTTCG	
**dCEN/NheI/F**	CGCGGCTAGCAAGACTCTAGGGGGATCGCCA	
**dCEN/NheI/R**	CGCGGCTAGCGAAAAGGACCCAGGTGGCACT	
**Y194/XhoI/0.9A/F**	CGCTCGAGATGGAACAACAGGCCCATG	The XhoI site is underlined
**Y194/BamHI/1.0A/F**	ATGTGGGATCCTTTACTCAACGAG	The BamHI site is underlined
**Y194/BamHI/R**	TAAAGGATCCCACATGTTTAACG	
**d-Y194-0.9Am1/F**	TCCGTATAATATCGCCGGA	The seventh and the eighth nucleotides (TA) of the nonamer are mutated into CG
**d-Y194-0.9Am1/R**	TCCGGCGATATTATACGGA	
**d-Y194-0.9Am2/F**	TTATTACATTTTGACTTAGTCAATTGG	Two stop codes (TAA) are inserted immediately after the C1 ATG start code
**d-Y194-0.9Am2/R**	CCAATTGACTAAGTCAAAATGTAATAA	
**d-Y194-0.9Am3/F**	TTCTTGATGTTGGACTTGACG	The 2288A mutated into T to make the C1^{Y103N} mutant
**d-Y194-0.9Am3/R**	CGTCAAGTCCAACATCAAGAA	
**d-Y194-0.9Am4/F**	TTATTACATTATCGTGACAGCCAATGG	Two stop codes (TAA) are inserted immediately after the C3 ATG start code
**d-Y194-0.9Am4/R**	CCATTGGCTGTCACGATAATGTAATAA	
**d-Y194-0.9Am5/F**	TTATTACATTACGATGTTTTTCTTGTG	Two stop codes (TAA) are inserted immediately after
**d-Y194-0.9Am5/R**	CACAAGAAAAACATCGTAATGTAATAA	

		the C4 ATG start code
**d-Y194-M1/F**	CCGCGGATCCATGGGTCACTGCATCTCCAT	The C4 ORF is inserted into dpRS316, the BamHI site is underlined
**d-Y194-M1/R**	GGAGCTCGAGAAGCTTTTAGGGCCTCTGCAGCAGCA	The XhoI and HindIII sites are underlined
**d-Y194-M2/F**	CCGCTCGAGCATTTTGACTTAGTCAATTGGGTG	The common region (CR) is inserted into dpRS316, the XhoI site is underlined
**d-Y194-M2/R**	CGCGGATCCGTTTAACGTAAATACTTGGGGCC	The BamHI site was underlined
**d-Y194-M3/F**	CGCAGGATCCAAGCTTATGCCTCGTCTTAATTCATTC	C1 ORF is inserted into dpRS316, the BamHI and HindIII sites are underlined
**d-Y194-M3/R**	CGCAGTCGACCTCGAGTCAACTCTCCGTCGTCTGG	The SalI and XhoI sites are underlined
**d-Y194-M4/F**	CCGCTCGAGTGTTTAGGGCCTCTGCAGC	The XhoI site is underlined
**d-Y194-M4/R**	TAAAGGATCCCACATGTTTAACG	The BamHI site is underlined
**d-Y194-M5/F**	CCGCTCGAGAGATCAACTCTCCGTCGTCTG	The XhoI site is underlined
**d-Y194-M5/R**	Identical to d-Y194-M4/R	Identical to d-Y194-M4/R
**d-Y194-M6/F**	CCGCTCGAGTACTTAAATACTCTTAAGAAACGC	The XhoI site is underlined
**d-Y194-M6/R**	Identical to d-Y194-M4/R	Identical to d-Y194-M4/R
**d-Y194-M9/F**	ATGTGGGATCCTTTACTCAACGAG	The BamHI site is underlined
**d-Y194-M9/R**	Identical to d-Y194-M3/R	Identical to d-Y194-M3/R
**d-Y194-M10/F**	Identical to d-Y194-M9/F	Identical to d-Y194-M3/F
**d-Y194-M10/R**	Identical to d-Y194-M1/R	Identical to d-Y194-M1/R
**d-Y194-M11/F**	CCGCTCGAGCCGTATCTATTTTTATGATTCTCAAATG	The XhoI site is underlined
**d-Y194-M11/R**	Identical to d-Y194-M4/R	Identical to d-Y194-M4/R
**d-Y194-M12/F**	Identical to d-Y194-M11/F	Identical to d-Y194-M11/F
**d-Y194-M12/R**	Identical to d-Y194-M2/R	Identical to d-Y194-M2/R

**d-Y194-M13/F**	Identical to d-Y194-M11/F	Identical to d-Y194-M11/F
**d-Y194-M13/R**	Identical to d-Y194-M3/R	Identical to d-Y194-M3/R
**d-Y194-M14/F**	Identical to d-Y194-M11/F	Identical to d-Y194-M11/F
**d-Y194-M14/R**	Identical to d-Y194-M1/R	Identical to d-Y194-M1/R
**d-Y194-M15/F**	Identical to d-Y194-M11/F	Identical to d-Y194-M11/F
**d-Y194-M15/R**	CGC<u>GGATCC</u>ACACAGGTTCCGCTGAAGCCGC	The BamHI site is underlined
**d-Y194-M16/F**	Identical to d-Y194-M11/F	Identical to d-Y194-M11/F
**d-Y194-M16/R**	CGC<u>GGATCC</u>GAATTCATGGGAGCCCAAAGGGAC	The BamHI site is underlined
**d-Y194-M17/F**	Identical to d-Y194-M11/F	Identical to d-Y194-M11/F
**d-Y194-M17/R**	CGC<u>GGATCC</u>ATGCGATCTTCGTCACCCTCCAAG	The BamHI site is underlined
**d-Y194-M18/F**	Identical to d-Y194-M11/F	Identical to d-Y194-M11/F
**d-Y194-M18/R**	CGC<u>GGATCC</u>TCTACCCTGTGGCTGTTCATAC	The BamHI site is underlined
**d-Y194-M19/F**	Identical to d-Y194-M11/F	Identical to d-Y194-M11/F
**d-Y194-M19/R**	CGC<u>GGATCC</u>ATGGATTCACGCACAGGGGAAC	The BamHI site is underlined
**d-Y194-M20/F**	Identical to d-Y194-M11/F	Identical to d-Y194-M11/F
**d-Y194-M20/R**	CGC<u>GGATCC</u>GTATCAATGTATTAAATATTTAGATAGATTG	The BamHI site is underlined
